# Selective blockade of rat brain T-type calcium channels provides insights on neurophysiological basis of arousal dependent resting state functional magnetic resonance imaging signals

**DOI:** 10.3389/fnins.2022.909999

**Published:** 2022-08-08

**Authors:** Vahid Khalilzad Sharghi, Eric A. Maltbie, Wen-Ju Pan, Shella D. Keilholz, Kaundinya S. Gopinath

**Affiliations:** ^1^Department of Biomedical Engineering, Emory University-Georgia Tech, Atlanta, GA, United States; ^2^Department of Radiology & Imaging Sciences, Emory University, Atlanta, GA, United States

**Keywords:** cortical slow wave activity, T-type calcium channel blocker, vigilance, arousal, animal studies, resting state fMRI, quasiperiodic patterns

## Abstract

A number of studies point to slow (0.1–2 Hz) brain rhythms as the basis for the resting-state functional magnetic resonance imaging (rsfMRI) signal. Slow waves exist in the absence of stimulation, propagate across the cortex, and are strongly modulated by vigilance similar to large portions of the rsfMRI signal. However, it is not clear if slow rhythms serve as the basis of all neural activity reflected in rsfMRI signals, or just the vigilance-dependent components. The rsfMRI data exhibit quasi-periodic patterns (QPPs) that appear to increase in strength with decreasing vigilance and propagate across the brain similar to slow rhythms. These QPPs can complicate the estimation of functional connectivity (FC) *via* rsfMRI, either by existing as unmodeled signal or by inducing additional wide-spread correlation between voxel-time courses of functionally connected brain regions. In this study, we examined the relationship between cortical slow rhythms and the rsfMRI signal, using a well-established pharmacological model of slow wave suppression. Suppression of cortical slow rhythms led to significant reduction in the amplitude of QPPs but increased rsfMRI measures of intrinsic FC in rats. The results suggest that cortical slow rhythms serve as the basis of only the vigilance-dependent components (e.g., QPPs) of rsfMRI signals. Further attenuation of these non-specific signals enhances delineation of brain functional networks.

## Introduction

Blood oxygenation level-dependent (BOLD) resting-state fMRI (rsfMRI) is a powerful tool for mapping the brain connectomes during normal function and illness (Bullmore and Sporns, 2009; Buckner et al., 2013). Numerous studies have shown that the performance in cognitive and sensory tasks is strongly influenced by the resting-state functional connectivity (FC) in relevant networks (Smith et al., 2009; Laird et al., 2011; Tavor et al., 2016). Impairments in brain FC networks have been shown to serve as biomarkers in a number of disease models (Greicius, 2008; Jalilianhasanpour et al., 2019). However, the neurophysiological basis underlying rsfMRI signals are not completely understood, which impedes the interpretation of these studies.

### Brain slow rhythms and resting-state functional magnetic resonance imaging (rsfMRI)

Several studies have surmised that rsfMRI signals are related to expression of cortical brain rhythms (He and Raichle, 2009; Scholvinck et al., 2010; Wang et al., 2012; Amemiya et al., 2016; Leong et al., 2016; Lu et al., 2016; Matsui et al., 2016; Wen and Liu, 2016; Chan et al., 2017), especially in gamma rhythms (Scholvinck et al., 2010; Liu et al., 2015a,b) and slow/delta rhythms (Lu et al., 2016; Matsui et al., 2016; Chan et al., 2017) frequency bands. Of particular interest are sub-1 Hz (∼0.1–1 Hz) fluctuations in delta rhythms (1–4 Hz) (Steriade et al., 1993a,b; Steriade, 2000; Crunelli and Hughes, 2010). From here onward, we refer to sub-1Hz waves as slow rhythms, and coherent oscillations in the 1–4-Hz band delta rhythms to avoid confusion. Slow rhythms share many properties with rsfMRI signals. They exist in the absence of stimulation, during wakefulness (Vyazovskiy et al., 2011; McCormick et al., 2015), sleep (Massimini et al., 2004; Crunelli and Hughes, 2010; Nir et al., 2011; David et al., 2013), and under sedation (Steriade et al., 1993b; Volgushev et al., 2006; Chauvette et al., 2011; David et al., 2013) and are influenced by vigilance/arousal (Steriade, 2000; Llinas and Steriade, 2006; Chauvette et al., 2011; McCormick et al., 2015), and propagate across the brain (Massimini et al., 2004; Sheroziya and Timofeev, 2014; Matsui et al., 2016). These properties are seen in rsfMRI signal patterns (Majeed et al., 2011; Mitra et al., 2015; Matsui et al., 2016; Yousefi et al., 2018; Liu and Falahpour, 2020) as well. In a series of studies (Leong et al., 2016; Chan et al., 2017; Wang et al., 2019), one group has shown that that low-frequency (e.g., 1 Hz) optogenetic stimulation of specific hippocampal and thalamic regions in rats evoked increased interhemispheric homotopic FC (IHFC) in various brain regions, whereas high-frequency stimulation of the same hippocampal region at 40 Hz had no effect on FCs (Chan et al., 2017; Wang et al., 2019), indicating that FC of rsfMRI signals may reflect neuronal dynamic related to slow rhythms. Another study in mice (Matsui et al., 2016) found that hemodynamic signals were closely associated with transient neuronal co-activations embedded in slow globally propagating waves of neural activity during rest, strengthening the view that the rsfMRI signals reflect slow wave activity. However, resting-state FC can in principle also be generated through other mechanisms such as gamma rhythms (Scholvinck et al., 2010) and spontaneous fluctuations in action potential (Ponce-Alvarez et al., 2015) between functionally connected brain regions. Importantly, unlike slow/delta rhythms, the strength of FC in canonical brain function networks generally decreases with reductions in vigilance and arousal levels (Larson-Prior et al., 2009; Hutchison et al., 2014; Bettinardi et al., 2015). Relatedly, a recent optical imaging study (Brier et al., 2019) indicated that the slow globally propagating neural activity in resting-state calcium and hemoglobin signals is separable from those that drive correlations between functionally connected brain regions. Another study (Okun et al., 2019) showed that the populations of neurons, which exhibit coherent fluctuations across the brain over slow and fast time-scales, are distinct from each other. Thus, a number of indicators exist that suggest rsfMRI signals encoding FC in brain functional and structural networks are distinct from fMRI signals reflecting slow rhythms. It is also possible that these rhythms serve as the basis of just the vigilance-dependent components of rsfMRI signals. For instance, the rsfMRI data exhibit quasi-periodic patterns (QPPs) wherein sets of cortical brain regions exhibit transient coherent activity (Thompson et al., 2014, 2015; Yousefi et al., 2018; Abbas et al., 2019). QPPs increase in strength with decreasing vigilance/arousal (Thompson et al., 2013a; Billings and Keilholz, 2018) and propagate across the brain (Majeed et al., 2009; Belloy et al., 2018) similar to brain slow rhythms. Another vigilance/arousal-dependent feature of rsfMRI signals is a transient cortex-wide neural signal component called global signal (Scholvinck et al., 2010; Falahpour et al., 2018) that induces correlations between voxel time-series across the brain that are not specific to any brain function networks. Global signal also increases with decreasing vigilance (Falahpour et al., 2018). In fact, some studies have shown that global signal is related to QPPs (Belloy et al., 2018). Both QPP and global signal complicate/confound the estimation of intrinsic FC in brain function networks.

### Role of T-type calcium channels in generation of slow rhythms

Given the contradictory hypotheses and findings regarding the relationship of BOLD rsfMRI signal and slow rhythms, a critical need exists to investigate the relationship between them. One way to do this is by examining the effects of systematically manipulating the expression of slow rhythms on rsfMRI signals. This can be achieved through pharmacological interventions. Although the slow rhythms are primarily generated through interaction of excitatory and inhibitory neurons in the cortex (Crunelli et al., 2015; McCormick et al., 2015), one mechanism for expression and maintenance of slow rhythms in the brain is through the interplay between cortical and thalamic slow rhythm generating oscillators (Crunelli et al., 2015). This mechanism is reviewed in detail in Crunelli and Hughes (2010) and Crunelli et al. (2015). In brief, cortical slow rhythms are characterized by long periods (“DOWN” states) of low-neural activity interspersed with “UP” states of increased excitability. The prolonged “UP” states of slow rhythms in cortical layer 5/6 neurons lead to long-lasting excitatory postsynaptic potentials (EPSPs) in thalamocortical neurons. These EPSPs induce slow rhythms in thalamic nuclei, which receive input from the cortical layer 5/6 neurons. The “UP” states in thalamus are preceded by a high-frequency burst generated by low-threshold T-type calcium (Ca^2+^) channels (TTCCs). These high-frequency bursts generate a new “UP” state of slow rhythms in cortical layer 4 neurons. This cycle of cortex to thalamus to cortex induction of UP and DOWN states helps maintain the rhythms. Slow waves in different thalamocortical circuits are not fully synchronized, which likely provide the basis for propagative properties of slow rhythms (Crunelli et al., 2015). A selective TTCC blocker (Dreyfus et al., 2010) TTA-P2 (3,5-dichloro-N-[1-(2,2-dimethyl-tetrahydro-pyran-4-ylmethyl)-4-fluoro-piperidin-4-ylmethyl]-benzamide), has been shown to inhibit this thalamic bursting activity, resulting in suppression of slow rhythms (Crunelli and Hughes, 2010; Crunelli et al., 2014). Selective blockade of voltage-gated Ca_v_3.1 TTCCs in first-order thalamic nuclei leads to significant reductions in the power of slow waves in corresponding primary cortices in rats (David et al., 2013). Systemic administration of the TTA-P2 induces an even greater (up to 60%) suppression of slow cortical oscillations (David et al., 2013) in rats, both during natural sleep and under anesthesia. TTA-P2 reversibly inhibits TTCCs but does not engage L-type calcium channels, sodium channels, or potassium channels and does not interfere with glutamatergic or GABAergic synaptic currents (Dreyfus et al., 2010). Hence, it provides ideal means for examining the effects of suppression of slow waves without affecting other forms of neural activity not related to TTCCs, e.g., tonic neuronal firing that subserve cognition (Sherman, 2001). Importantly, though TTA-P2 administration suppresses slow rhythms, it paradoxically increases somnolence in rats (Kraus et al., 2009; McCafferty et al., 2012; Crunelli et al., 2014). TTA-P2 does not have any renal or cardiovascular effects (Shipe et al., 2008), and it does not affect arterial blood flow (Masicampo et al., 2018). Hence, it is not likely to induce global changes in cerebral blood flow.

In this preliminary study, we examined the effects of TTA-P2 on rsfMRI signals in rodents. Based on the studies described above, we hypothesized that systemic administration of TTA-P2 will induce significant decrease in the strength of rsfMRI QPPs. And this reduction in the strength of signals, not specific to brain function networks, will increase rsfMRI estimates of intrinsic FC, thereby enhancing the power of rsfMRI technique to probe brain function networks.

## Materials and methods

### Rodents and preparation

All protocols for animal studies were reviewed and approved by the Emory University Institutional Animal Care and Use Committee (IACUC) and were in compliance with the NIH guidelines. Twelve Sprague-Dawley rats (male, 300–350 g) were employed in this study. Seven rats were administered the TTCC TTA-P2, and five were administered the vehicle. Animal preparation and imaging were conducted with long established techniques (Pan et al., 2013; Thompson et al., 2014, 2015) and more details are provided in Supplementary materials. The fMRI data were collected under dexmedetomidine (Dexmed) anesthesia using long-established techniques (Thompson et al., 2013b,^2014^, ^2015^). Dexmed was administered in the form of a 0.025-mg/kg bolus and maintained under sedation with a constant subcutaneous (s.c.) infusion of 0.05 mg/kg/h. Pancuronium bromide was simultaneously administered at a constant rate of 1 mg/kg/h to minimize animal motion. Body temperature, respiration rate, end-tidal CO_2_, blood oxygen saturation (SpO_2_), and heart rate were all continuously monitored. After acquiring rsfMRI data under these conditions for 20–90 min, seven rats were injected (s.c.) with 6 mg/kg TTA-P2 dissolved in 2.5 ml of vehicle (4% dimethyl sulfoxide (DMSO) saline) and studied for another 60–90 min. Similarly, data from 5 other rats were acquired with just the vehicle control.

### Magnetic resonance (MR) imaging

The MRI data were acquired in a 9.4-T Bruker MRI scanner under Dexmed anesthesia regime as described above. A custom-made oval surface transceiver radiofrequency (RF) coil with an internal diameter of 2 cm × 2.5 cm (Pan et al., 2021) was placed directly over the rat brain to acquire MRI signals. The fMRI scans were acquired with a gradient-echo EPI sequence with the following parameters: TR = 2,000 ms, TE = 20 ms, flip angle = 60°, matrix size 70 × 70, field of view 3.45 cm × 3.45 cm. Twenty-four 0.5-mm thick coronal slices covering the almost the entire brain (see Supplementary Figure 1). A 2D fast spin echo MRI scan (TR/TE = 3,500 ms/44 ms; 24 coronal slices) was also acquired with the same resolution and coverage as the EPI scans.

### Data analysis

#### Functional magnetic resonance imaging (fMRI) data preprocessing

An fMRI data preprocessing was conducted with FSL (Smith et al., 2004) and AFNI (Cox, 1996) tools, and in-house MATLAB scripts. The rsfMRI voxel time-series were corrected for magnetic susceptibility-induced geometric distortions (Andersson et al., 2018), temporally shifted to account for differences in slice acquisition times and 3D volume registered to a base volume to account for global rigid motion. These time-series were co-registered to the T2-weighted high-resolution anatomic scan, and spatially normalized to the Paxinos atlas template (Valdes-Hernandez et al., 2011; Paxinos and Watson, 2014), with the warp computed from alignment of the high-resolution T2-weighted anatomic to the Paxinos atlas template. The spatially normalized fMRI time-series were bandpass (0.01–0.20 Hz) filtered, detrended of similarly filtered estimated motion parameters time-courses through linear regression, and standardized. The resultant time-series were spatially smoothed with an isotropic Gaussian filter (FWHM = 1 mm).

#### Quasi-periodic pattern (QPP) analysis

Since global signal regression (GSR) enhances the delineation of QPPs (Yousefi et al., 2018; Abbas et al., 2019), the whole-brain average fMRI signal was filtered (0.01–0.20 Hz) and regressed out during the preprocessing fMRI time-series (see Section “Functional magnetic resonance imaging (fMRI) data preprocessing”). Finally, QPPs were extracted from the concatenated pre-injection (Baseline) of all rats’ fMRI data. Supplementary Figure 2 illustrates the process of identifying the expression of a QPP from a representative rsfMRI time-series dataset. Briefly, the QPP algorithm (Majeed et al., 2011) randomly selects an epoch (initial guess for a QPP template) of spatiotemporal data (5- to 10-s duration) and calculates sliding-window correlation between the template and the entire time-course, identifying time points of high correlation (with a set threshold arrived at with bootstrapping; see Majeed et al., 2011). The spatiotemporal blocks centered on these time points are then averaged to create a revised spatiotemporal template of that QPP, and this process is repeated until the template converges. The changes in the strength of the expression of QPPs over time for each fMRI scan (Baseline and TTA-P2) for each rat were estimated through the sliding window spatiotemporal correlation (STC) of the corresponding fMRI time-series with the rats’ QPP template (Majeed et al., 2011). The effects of TTA-P2 on QPPs were assessed with between-session (TTA-P2 vs. Baseline) paired *t*-tests on the mean of positive excursions of the STC curve above zero. Similar analysis was performed to assess differences between Vehicle and Baseline sessions for the five rats, which were only administered the vehicle.

#### Interhemispheric homotopic functional connectivity (FC) analysis

The FCs in intrinsic brain function networks were assessed by examining the FC between homotopic brain regions in the two hemispheres, since these are in general known to possess high FC (Stark et al., 2008; Shen et al., 2015; Wang et al., 2019). Further IHFC has been employed as a measure of intrinsic FC in some recent studies examining the neurophysiological basis of rsfMRI, which implicates slow rhythms as a likely basis for intrinsic FC (Matsui et al., 2016; Chan et al., 2017). To examine the effects of slow wave suppression on fMRI IHFC, the preprocessed time-series (see Section “Functional magnetic resonance imaging (fMRI) data preprocessing”) was broken down into 10-min segments (in order to examine any systematic evolution of the FC over time). Subsequently, region of interest (ROI) average reference vectors were obtained from the 48 homotopic brain regions that were labeled in the Paxinos atlas space in a well-established rat MRI template (Valdes-Hernandez et al., 2011). IHFC for each region (for a given 10-min segment) was assessed during Baseline and TTA-P2/Vehicle sessions through the cross-correlation coefficient (CC) between the left and right hemisphere ROIs of the corresponding regions in a general linear regression framework (Cox, 1996). Since the dynamic effects of TTA-P2 on FC were found to be transient and variable across the rats, the 10-min segment for each rat where most of the homotopic ROIs showed maximum FC was selected as the one where the effects of TTA-P2 was strongest across the brain. Since all the 10-min segments in the Baseline condition are equivalent, Baseline IHFC was arrived at by taking the median of the IHFC in all 10-min segments. The effects of TTA-P2 (and Vehicle) were assessed through paired *t*-test on the IHFC of different brain regions. Multiple-comparison correction was conducted through false discovery rate (FDR) (Benjamini, 2010). Further reproducibility of TTA-P2 effects across the rats for each ROI was assessed *via* the number of rats, which exhibited increased IHFC after TTA-P2 administration as a fraction of the total number of rats.

#### Thalamocortical functional connectivity (FC) analysis

Since the mechanism of slow rhythms suppression involves blockade of thalamic TTCCs, the FCs in different thalamocortical networks were estimated through the CC between ipsilateral Paxinos ROIs of primary somatosensory, motor, auditory and visual cortex and their corresponding thalamic centers (ventrobasal (VB) nucleus, ventrolateral (VL) nucleus, medial geniculate nucleus (MGN), and lateral geniculate nucleus (LGN) ROIs). Similarly, IHFC of these four first-order thalamic nuclei and intrathalamic FC between them were also obtained. TTA-P2 effects on FC were assessed as described above (Section “Interhemispheric homotopic functional connectivity (FC) analysis”). Intrathalamic FC were assessed between LGN and the ventrobasal (VB) nucleus complex, and between MGN and ventrolateral (VL) nucleus. This was because VB and VL nuclei, and LGN and MGN are adjacent, which could lead to overlap between some of their fMRI voxels, and vasculature. The thalamic nuclei ROIs were obtained using a publicly available atlas (Papp et al., 2014).

#### Seed-based cross-correlation maps

Finally, the whole-brain FC maps of the rat barrel cortex (S1BF) and auditory cortex [both of which are unimodal regions, which are known to possess high IHFC (Stark et al., 2008)], were obtained through CC between all voxels in the brain and the right S1BF and right auditory cortex seed ROI average fMRI time-series vectors, for all 10-min fMRI segments of each session. TTA-P2/Vehicle vs. Baseline differences in the FC group paired *t*-tests are as described above (Section “Interhemispheric homotopic functional connectivity (FC) analysis”). Statistical inference test maps were clustered, and the significance of activations, accounting for multiple comparisons, was derived by means of Monte Carlo simulation of the process of image generation, spatial correlation of voxels, intensity thresholding, masking, and cluster identification (Cox et al., 2017; Gopinath et al., 2018).

## Results

None of the rats exhibited significant motion (i.e., frame-to-frame displacement was always less than 0.02 mm). Furthermore, no meaningful changes were observed in the physiological signals monitored: respiration rate, hear rate, oxygen saturation, and body temperature between Baseline and TTA-P2/Vehicle sessions.

### TTA-P2 administration reduced strength of quasi-periodic patterns (QPPs)

**FIGURE 1:**
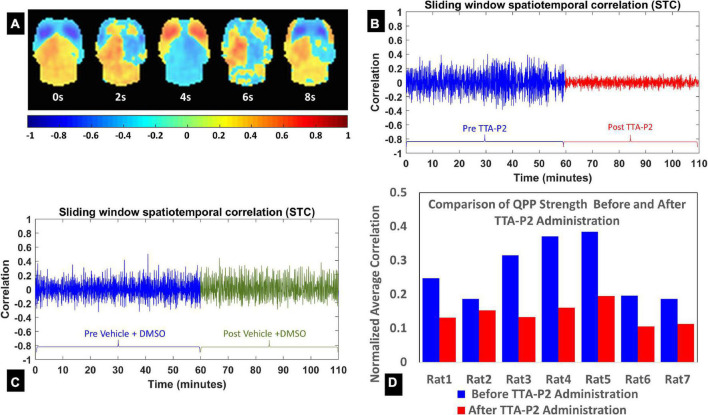
**(A)** Eight frames from the quasi-periodic pattern (QPP) template obtained from both groups’ pre-injection concatenated functional time-series (window length = 10 s). **(B,C)** The evolution of the QPP strength with time assessed with spatiotemporal correlation of the fMRI time-series with corresponding QPP template on one rat **(B)** after systematic administration of TTA-P2; and one rat **(C)** after systematic administration of Vehicle. **(D)** The QPP strength changes for each rat in the TTA-P2 group. The values are estimated as the mean of positive excursions of the spatiotemporal correlation (STC) curve above zero, normalized by the maximum correlations for each subject.

Figure 1A shows a QPP with anterior-to-posterior propagation. The fluctuation in the strength of the QPP before and after injection of TTA-P2 is shown for a representative rat administered TTA-P2 (Figure 1B), and one administered vehicle (Figure 1C). TTA-P2 administration significantly (paired *t*-test, *p* < 0.002) reduced the strength (mean of positive STC values) of QPPs compared to Baseline. All the rats exhibited suppression of QPPs after TTA-P2 administration. The amount of suppression of QPPs induced by TTA-P2 varied from 18 to 58% (mean 48%) across the rats (see Figure 1D). Performing QPP analysis without GSR (Supplementary Figure 3) reduced the significance of TTA-P2 vs. Baseline differences to paired *t*-test, *p* < 0.005. This analysis yielded 8–37% (mean 27%) suppression in QPPs after TTA-P2 administration. Vehicle did not appreciably alter the strength of the QPPs, with or without GSR.

### TTA-P2 administration increased interhemispheric homotopic functional connectivity (IHFC)

**FIGURE 2:**
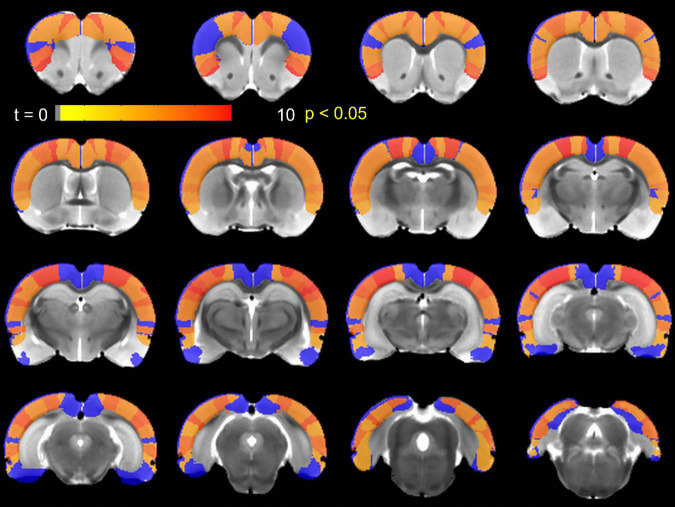
Paxinos region of interest (ROIs) exhibiting significantly (*p* < 0.05) enhanced interhemispheric homotopic functional connectivity (IHFC) relative to Baseline after TTA-P2 injection overlaid on a Paxinos atlas brain. ROIs shown in blue did not achieve significance.

Next, we examined IHFC of the Paxinos atlas regions. This Paxinos template (Valdes-Hernandez et al., 2011) has 48 homotopic ROIs (as listed in Supplementary Table 1), but 8 of these ROIs did not yield any fMRI signal in one or both hemispheres. Thirty-one of the remaining forty ROIs exhibited (Figure 2) significantly [paired *t*-test, *p* < 0.05 (Table 1)] increased IHFC after injection of TTA-P2. FDR correction rendered TTA-P2 vs. Baseline differences in IHFC in 4 out of these 31 ROIs above significance (FDR q = 0.05) after accounting for multiple comparisons. Reproducibility of the IHFC increases after TTA-P2 administration varied between 86% and 100% (mean 93%) among the IHFCs of the ROIs, which exhibited significant increase in IHFC after TTA-P2 administration (Supplementary Figure 4). Analyzing IHFC after GSR yielded TTA-P2 engendered enhancements in 22 ROIs after FDR correction (Supplementary Table 2). Vehicle administration did not evoke appreciable (significant) changes in IHFC.

**TABLE 1:** TTA-P2 vs. baseline interhemispheric homotopic functional connectivity (IHFC) paired *t*-test for different Paxinos region of interest (ROIs).

Paxinos ROI	*t*	*p*	FDR *p*	Paxinos ROI	*t*	*p*	FDR *p*
AID right	4.209	0.006	0.02	PtPD right	4.038	0.007	0.021
AIP right	2.57	0.042	0.056	PtPR right	4.443	0.004	0.02
AIV right	6.048	0.001	0.017	RSD right	2.011	0.091	0.104
APir right	NA	NA	NA	RSGb right	1.079	0.322	0.322
Au1 right	3.29	0.017	0.036	RSGc right	1.597	0.161	0.17
AUD right	4.266	0.005	0.02	S1 right	5.687	0.001	0.017
AuV right	2.126	0.078	0.091	S1BF right	3.277	0.017	0.036
Cg1 right	3.056	0.022	0.039	S1DZ right	3.002	0.024	0.039
Cg2 right	3.192	0.019	0.038	S1DZ0 right	1.805	0.121	0.131
DI right	3.258	0.017	0.036	S1FL right	3.944	0.008	0.022
DIEnt right	NA	NA	NA	S1HL right	4.796	0.003	0.02
DLEnt right	2.544	0.044	0.057	S1J right	1.918	0.104	0.115
DLO right	2.623	0.039	0.054	S1Sh right	NA	NA	NA
Ect right	3.821	0.009	0.023	S1Tr right	5.168	0.002	0.02
Fr3 right	2.289	0.062	0.078	S1ULp right	3.047	0.023	0.039
GI right	2.891	0.028	0.041	S2 right	3.562	0.012	0.03
GIDI right	NA	NA	NA	TeA right	2.633	0.039	0.054
LPtA right	4.809	0.003	0.02	V1 right	6.126	0.001	0.017
M1 right	2.956	0.025	0.039	V1B right	3.124	0.02	0.039
M2 right	4.427	0.004	0.02	V1M right	4.026	0.007	0.021
MEnt right	NA	NA	NA	V2L right	4.277	0.005	0.02
MPtA right	2.21	0.069	0.084	V2ML right	2.967	0.025	0.039
PRh right	NA	NA	NA	V2MM right	1.458	0.195	0.2
PtPC right	NA	NA	NA	VIEnt right	NA	NA	NA

dof, degrees of freedom; FDR *p*, false discovery rate corrected *p*; *see* Supplementary materials for Paxinos ROI abbreviations.

### TTA-P2 administration did not affect thalamocortical functional connectivity (FC)

The FC between primary sensory and motor cortices and their corresponding first-order thalamic nuclei were not significant (1-sample *t*-tests, *p* > 0.05), both in Baseline and TTA-P2 sessions. TTA-P2 administration did not have appreciable effect on these connections. The IHFC of these thalamic nuclei, and FC between VB and LGN thalamus, and between MGN and VL thalamus increased with TTA-P2 administration, but the results were not significant at paired *t*-test, *p* < 0.05. However, these intrathalamic FCs were significantly strong (1-sample *t*-tests, *p* < 0.05) in both sessions. GSR did not change the results appreciably.

### TTA-P2 administration increased functional connectivity (FC) in canonical brain function networks

**FIGURE 3:**
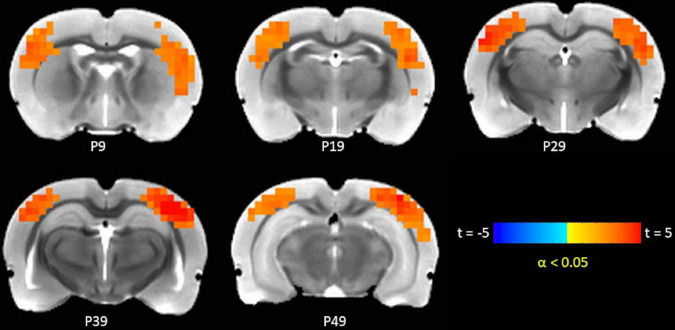
TTA-P2 vs. Baseline *t*-statistic maps highlighting regions with enhanced functional connectivity (FC) to right S1BF ROI after TTA-P2 administration. The slice-location coordinates are in Paxinos space. Left hemisphere is on the left-hand side of the maps.

**FIGURE 4:**
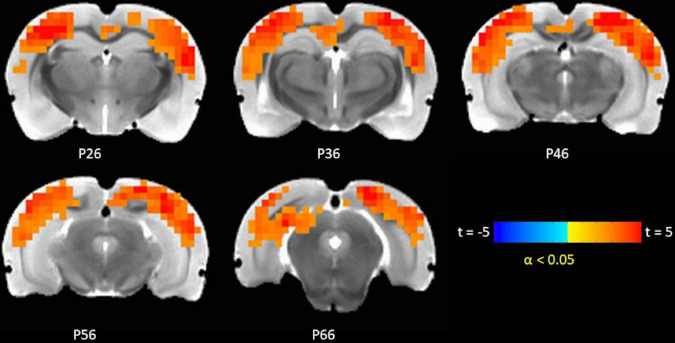
TTA-P2 vs. Baseline *t*-statistic maps highlighting regions with enhanced functional connectivity (FC) to right auditory cortex region of interest (ROI) after TTA-P2 administration. The slice-location coordinates are in Paxinos space. Left hemisphere is on the left-hand side of the maps.

The TTA-P2 significantly (paired *t*-test, FWE α < 0.05) increased the rsfMRI FC between right S1BF and some areas in somatosensory, motor, auditory, visual, and parietal cortices bilaterally (Figure 3). Right auditory cortex exhibited significantly increased FC to contralateral auditory, visual, and somatosensory areas (Figure 4). Both seed ROIs exhibited strong FC with most of the above functionally connected regions in the Baseline session (Supplementary Figures 5, 6). These effects persisted after GSR (Supplementary Figures 7, 8), but the extent and strength of the TTA-P2 vs. Baseline FC differences decreased. Vehicle administration did not evoke appreciable changes in FC.

## Discussion

### Context and rationale for study

Although 20–40% of the rsfMRI signal can be attributed to vasomotion, and physiological noise, there remains a large portion of the rsfMRI signal that can be attributed to neural signals (Mayhew et al., 1996; Biswal et al., 1997; Mitra et al., 1997; Birn et al., 2006; Bianciardi et al., 2009). The neurophysiological basis of these signals are not clear with the first studies directly relating rsfMRI signal to neural activity published not long ago (Leopold et al., 2003; Nir et al., 2008). Some groups have hypothesized that rsfMRI signal underlying FC in brain function networks could arise from spontaneous neural activity in these networks in the absence of any tasks (Chan et al., 2015; Ponce-Alvarez et al., 2015). On the other hand, many groups have observed correlations between fluctuations in the power of different brain rhythms obtained from electrophysiological recordings and BOLD fMRI signal (Scholvinck et al., 2010; Pan et al., 2013; Liu et al., 2015b; Lu et al., 2016). However, it must be noted that these observations do not clarify whether fMRI signal encode all of the neuronal dynamics that compose brain rhythms (Matsui et al., 2016), or discrete events within or driving these rhythms (Schwalm et al., 2017). It must be noted that different components of the rsfMRI signal could reflect different neural phenomena. For instance, fMRI signals linked to slow rhythms could be distinct from those that serve as the basis for rsfMRI FC in canonical brain function networks. In contrast with slow rhythms, the strength of FC in resting-state brain function networks generally decreases with reductions in vigilance and arousal levels (Larson-Prior et al., 2009; Hutchison et al., 2014). Relatedly, results from a few recent studies (Brier et al., 2019; Okun et al., 2019) indicated that hemodynamic signals related to globally propagating slow waves of neural activity are distinct from those that induce correlations between functionally connected brain regions. Hence, we hypothesized that only the arousal/vigilance-dependent components of rsfMRI signals arise from neural events related to expression of slow rhythms. And removing these rsfMRI signal components will enhance the delineation of brain function networks.

### TTA-P2 attenuates functional magnetic resonance imaging (fMRI) quasi-periodic patterns

The fMRI data exhibit QPPs wherein sets of cortical brain regions exhibit transient coherent activity (Majeed et al., 2009, 2011). These patterns propagate across the brain similar to slow rhythms brain (Majeed et al., 2009; Belloy et al., 2018). In addition, just like slow rhythms, QPPs increase in strength with decreasing vigilance/arousal (Thompson et al., 2013a; Billings and Keilholz, 2018), QPPs also complicate the estimation of FC with rsfMRI, either by existing as unmodeled signal or by inducing additional wide-spread correlation between voxel time-courses of functionally disparate brain regions. To examine the dependence of QPPs on slow rhythms, we employed a well-established pharmacological model, which has been shown to reproducibly suppress slow rhythms in rats (Dreyfus et al., 2010; Crunelli et al., 2015). As described in “Role of T-type calcium channels in generation of slow rhythms,” slow waves are maintained through the interplay between cortical and thalamic slow rhythm generating oscillators (Crunelli and Hughes, 2010; Crunelli et al., 2015). Importantly, the initiation of UP states of slow rhythms in thalamus is preceded by a TTCC-mediated high-frequency burst of thalamocortical EPSPs that initiate a new UP state of slow rhythm in the cortex. Administration of the TTCC blocker TTA-P2 suppresses this high-frequency burst of thalamocortical EPSPs, thereby suppressing slow rhythms (Dreyfus et al., 2010; David et al., 2013). In our experiment, systemic administration of TTA-P2 led to significant attenuation in the strength of the QPPs. These results indicate that *putative* suppression of slow rhythms diminishes the strength of QPPs, thereby reducing the arousal/vigilance-dependent contributions to fMRI signal. This effect was observed in all seven rats, which were administered TTA-P2 in this experiment, indicating that it is very reproducible. The suppression of QPPs occurred almost immediately after TTA-P2 administration. The absence of electrophysiological recordings time-locked to the fMRI signal in this study rendered it impossible to estimate relationship between QPPs and slow rhythms. However, one group (Schwalm et al., 2017) has shown cortex-wide correlations between fMRI signal and onset of cortical slow waves. This indicates that the global increases in signal often seen with transition between vigilance/arousal states (Liu et al., 2015a; Liu and Falahpour, 2020) could be related to the high-frequency burst of EPSPs that thalamus sends to the cortex at onsets of slow wave UP states. Similarly, QPPs could be related to the propagation of these slow wave onsets across the cortex (Crunelli et al., 2015). The fact that the strength of both global signal and QPPs are low during wakefulness could be related to the decreased frequency of thalamic bursting events, and hence onsets of slow wave UP states during wakefulness (Sherman et al., 2006; Vyazovskiy et al., 2011).

### TTA-P2 enhances functional connectivity (FC) in brain function networks

In addition to suppression of QPPs, TTA-P2 administration led to increased IHFC between homotopic cortical ROIs, especially those belonging to unimodal regions (e.g., primary sensory and motor cortices), which have been shown to possess highest IHFCs in the brain (Stark et al., 2008). Nine Paxinos cortical ROIs did not exhibit significant increase in IHFC after TTA-P2 administration. Seven of these nine ROIs were in heteromodal regions (frontal and associative cortices), which generally do not exhibit strong IHFC in rsfMRI studies (Stark et al., 2008; Shen et al., 2015). The remaining two were very small ROIs, thereby more susceptible to noise. The reproducibility of these results was not as strong as the suppression of QPPs (Supplementary Figure 4). Reproducibility varied between 86 and 100% (mean 93%) among the IHFCs of the ROIs, which exhibited significant increase in IHFC after TTA-P2 administration. This lack of perfect reproducibility could be due to variations in effects of anesthesia and/or the drug across the rats. The effects of the drug were variable with some rats exhibiting immediate decreases in IHFC after TTA-P2 injection, whereas in some cases, IHFC increases were more delayed.

Similar to IHFC, intrinsic FC in brain areas functionally connected to rat barrel and auditory cortices also increased after TTA-P2 administration. TTA-P2 significantly increased the rsfMRI FC between right S1BF and some areas in somatosensory, motor, auditory, visual, and parietal cortices bilaterally. Anatomical and functional connections between rat barrel cortex and these brain areas have been observed in a number of studies (Frostig et al., 2008; Bedwell et al., 2014; Zakiewicz et al., 2014). Right auditory cortex exhibited significantly increased FC to contralateral auditory, visual, and somatosensory areas, consistent with prior functional and anatomic tracing studies (Smith et al., 2010; Schormans et al., 2016; Thomas et al., 2020). Thus, the suppression of arousal-dependent fMRI signals (QPPs) by TTA-P2 increases apparent intrinsic FC in cortical brain function networks as hypothesized.

Finally, thalamocortical FC between first-order thalamic nuclei and cortical regions to which they project to did not change appreciably after TTA-P2 administration. However, the strengths of thalamocortical FC in both Baseline and TTA-P2 sessions were also low. Studies have shown that the light anesthetic regimen employed in our study could be analogous to slow wave sleep, both in terms of the slow rhythms (Torao-Angosto et al., 2021) and FC patterns (Greicius et al., 2008; Bettinardi et al., 2015). Thalamus can be decoupled from cortex (in terms of tonic thalamic activity) during some general anesthesia conditions (Suzuki and Larkum, 2020), and thalamocortical connectivity is often reduced during anesthesia and deep sleep (Esser et al., 2009; Hudetz, 2012; Picchioni et al., 2014). On the other hand, IHFC of thalamic nuclei and intrathalamic FC, both increased after TTA-P2 administration, though the differences were not significant (paired *t*-test, *p* > 0.05). Furthermore, both IHFC and intrathalamic FC were significant and strong in both Baseline and TTA-P2 session. Since there are no interhemispheric homotopic monosynaptic connections between these thalamic nuclei, this indicates that the strong rsfMRI IHFC observed between them is polysynaptic in nature. Interestingly, thalamic fMRI signal has been observed to decrease with arousal/vigilance in contrast to cortical global signal and QPPs in many studies (Chang et al., 2016; Liu and Falahpour, 2020). Blockade of TTCCs suppress slow rhythms in thalamus along with the cortex. However, thalamic signal increased in our study after TTA-P2 administration (i.e., slow wave suppression). Hence, decreases observed in thalamic signal with vigilance/arousal (Chang et al., 2016; Liu and Falahpour, 2020) are likely not related to slow rhythms.

### Limitations and pitfalls

Since this was a preliminary study, there are still a lot of unresolved issues. The major drawback of this study is that electrophysiological data were not acquired during the fMRI scans. Hence, there is no direct evidence that cortical slow rhythms were attenuated by TTA-P2. Although a well-established pharmacological model (David et al., 2013) for slow wave suppression was employed, lack of electrophysiological recordings time-locked to the fMRI data hindered both confirmation of slow wave suppression and ability to obtain quantitative relationships between slow rhythms and fMRI QPPs. Furthermore, although strength of QPPs decreased after TTA-P2 administration in all rats, the magnitude of suppression of QPPs varied significantly across the rats. In addition, the dynamic changes in IHFC with time exhibited large variations across the rats in both Baseline and TTA-P2 sessions. Obtaining simultaneous electrophysiological data could have helped to separate the variabilities that could be attributed to drug and anesthetic effects.

Second, although physiological parameters: respiration rate, end-tidal CO_2_, hear rate, oxygen saturation, and body temperature were monitored, these data could not be digitally recorded and time-locked to the fMRI data. Observationally, no substantial changes were observed in these physiological responses between Baseline and TTA-P2 sessions, which is consistent with previous studies (Shipe et al., 2008; David et al., 2013). However, the contributions of these physiological responses, which are known to affect BOLD fMRI data (Birn et al., 2006; Chang et al., 2009), could not be regressed out. Physiological noise was minimized before estimation of QPPs with GSR. Performing QPP analysis without GSR did not change the conclusion that QPP strengths were significantly attenuated upon TTA-P2 administration. The FC results presented in Figures 2–4 were obtained *without* GSR due to well-known confounds (Murphy et al., 2009) during estimation of correlations. Analyzing seed ROI based FC after GSR yielded TTA-P2 engendered IHFC enhancements in only 22 ROIs after FDR correction (Supplementary Table 2). However, GSR did not affect the maps of TTAP2 vs. Baseline paired *t*-tests in FC to right S1BF and right auditory cortices ROIs significantly (see Supplementary Figures 7, 8).

Ideally, the rats should be scanned when they are awake. However, this was not feasible within the constraints of this study. Slow rhythms are suppressed by TTA-P2 under even deep levels of anesthesia in the studies (David et al., 2013) validating the pharmacological model employed in this project. These studies employed xylazine and ketamine, whereas Dexmed and pancuronium bromide were employed in this project. At the low levels of anesthesia employed in this study, the two drug combinations should have similar effects. FC networks have been reliably obtained under both these anesthesia regimens in rats (Hutchison et al., 2010; Magnuson et al., 2014). However, Dexmed anesthesia best approximates natural sleep, in terms of its electrophysiological signatures and sensitivity to slow rhythms among different classes of anesthesia including isoflurane and ketamine (Musizza et al., 2007; Akeju and Brown, 2017). Furthermore, we could not employ isoflurane in this study as it can affect the action of the drug TTA-P2 (Joksovic et al., 2005).

The fMRI cortico-cortical FC decreases but is by and large intact in general going from wakefulness to the low levels of anesthesia employed in this project (Hutchison et al., 2014; Magnuson et al., 2014; Bettinardi et al., 2015). This is consistent with the results of this project. Similarly, arousal-dependent fMRI signal and slow rhythms, while reduced, are also present during light anesthesia and wakefulness. Hence, results of our project are translatable to common fMRI studies conducted while subjects are awake.

Finally, the sample size of this preliminary study was small, which reduced the effect size. Hence, the experiments will need to be repeated with larger sample sizes to confirm these results. All of these above limitations will be eliminated in future studies planned for this project.

## Conclusion

The results indicate that the vigilance-dependent components of the rsfMRI signal (e.g., QPPs) reflect the dynamics of cortical slow rhythms. Suppression of slow rhythms reduces the strength of vigilance-dependent rsfMRI signals and enhances intrinsic FC derived *via* rsfMRI in canonical brain function networks. These results have profound implications to our understanding of neurophysiological basis of rsfMRI signals. Future work would include large sample sizes, and simultaneous EEG/optical imaging recordings to directly examine cortical slow rhythms, and intrathalamic administration of TTA-P2 to specifically target only those TTCCs that are part of thalamocortical slow wave generating unit.

## Data availability statement

The raw data supporting the conclusions of this article will be made available by the authors, without undue reservation.

## Ethics statement

The animal study was reviewed and approved by Emory University Institutional Animal Care and Use Committee.

## Author contributions

VK conducted all the experiments in the project including imaging and data analysis. EM conducted some of the data analysis. W-JP provided critical advice in setting up the experiment. SK conceived the idea, developed the experiment and supervised VK, EM, and W-JP. KG conceived the idea, developed the experiment conducted data analysis and wrote the manuscript. All authors contributed to the article and approved the submitted version.
