# Supplementary material for: Selective blockade of rat brain T-type calcium channels provides insights on neurophysiological basis of arousal dependent resting state functional magnetic resonance imaging signals
Source: Front Neurosci. 2022 Aug 8;16:909999. doi: 10.3389/fnins.2022.909999 (PMC9393715; doi:10.3389/fnins.2022.909999)
Supplement: Supplementary file 1 [file Data_Sheet_1.pdf]

Supplementary Materials

Supplementary Methods: Rodents & Preparation

All protocols for animal studies were reviewed and approved by the Emory University Institutional Animal Care and Use Committee (IACUC) and were in compliance with NIH guidelines. Twelve Sprague-Dawley rats (male, 300-350g) were employed in this study. Seven rats were administered the TTCC TTA-P2, and five were administered the vehicle. All rats were housed in the Emory University's animal facility and were checked regularly by the investigators and by members of the clinical veterinary staff regarding their health and to recognize any signs of distress. The rats were euthanized at the end of experiments. After preparing the animal cradle and connecting water bath lines and physiological monitoring systems, the animal was weighed and then transferred to the anesthesia chamber. The rat was anesthetized using 5% isoflurane for 5 min which is typically sufficient to induce deep anesthesia. A heating pad was used under the anesthesia chamber to maintain the animal's body temperature at around 37 C°. Rat's physiological responses were monitored for signatures of deep anesthesia. Then animals' toes were pinched to ensure the animal is fully anesthetized. At this point, the gas flow was switched to the intubation platform and reduce to 2% isoflurane. The rat was carefully intubated using an intubation needle, which was then connected to a volume-cycled ventilator (Small Animal Ventilator, Model SAR-830/AP; CWE Inc., Ardmore, PA, USA). The integrity of intubation was confirmed with a capnograph (V90041, Surgivet, Inc., Waukesha, WI, USA), The animal was transferred to the cradle and the 2% isoflurane flow was redirected to the cradle. The ventilation lines and body/neck position were checked to ensure that SpO2 >95%. A pulse oximeter was attached to the sole of the hind paw. A rectal thermometer probe was lubricated and positioned to monitor body temperature accurately. Two 25g needles were placed in the rat for subcutaneous

injection of dexmedetomidine (Dexmed) and pancuronium into right and left thighs respectively. The Dexmed and pancuronium lines were connected to two syringe infusion pumps (KD Scientific, Holliston, MA) located outside of the magnet room. Subsequently, two 25g needles were inserted bilaterally on the rat dorsum on either side of the spine midway between lumbar cervical region, for subcutaneous injection of TTA-P2. The needles were attached (using tubing of similar diameters) to two 5mL syringes which were secured to either side of the cradle and which stayed outside the magnet bore. The water bath feeding the water lines of the cradle was adjusted to maintain a body temperature at 37º ± 0.5ºC. The rat's head was secured by two ear-bars (at a site frontal to the ear canal) as well as a tooth-bar to keep the head level and oriented along the center line of the cradle. This ensures that no head-movement occurs with respiration and other sources of motion. A surface transceiver RF coil was placed directly over the rat brain and secured using a set of grooves and screws to acquire MRI signals. A Dexmed bolus of 0.025 mg/kg was administered subcutaneously to the animal. After 5 minutes Dexmed was continuously administered with an infusion pump at a constant rate of 0.05 mg/kg/hr. Pancronium was simultaneously administered subcutaneously at constant rate of 1 mg/kg/hr using a second infusion pump. Five minutes after start of continuous Dexmed infusion, isoflurane was discontinued, and the animal was only anesthetized using Dexmed. Subsequently, the cradle was placed in the scanner ensuring that intended volume is exactly in the center of the magnet bore.

Supplementary Methods: Spatial Coverage of FMRI

FMRI scans were acquired with a gradient-echo EPI sequence with the following parameters: TR= 2000 ms, TE = 20 ms, flip angle = 60°, matrix size 70 X 70, field of view 3.45 cm X 3.45

cm. Twenty-four 0.5-mm thick coronal slices covering the almost the entire brain (see Supplementary Figure 1).

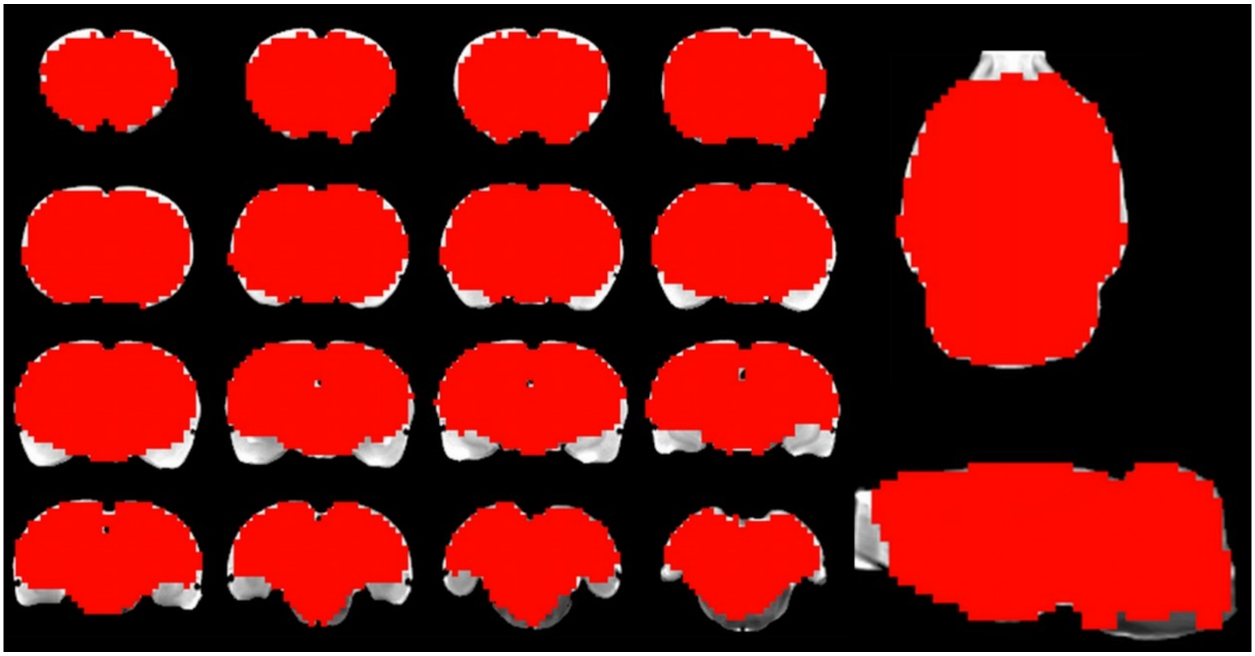

Supplementary Figure 1: Common group mask of all rats' spatially normalized EPI scans (in red) overlaid on the Paxinos Atlas Brain. The imaging parameters covered almost all but not the whole brain.

Supplementary Methods: QPP Extraction:

Our QPP algorithm (63) randomly selects an epoch (initial guess for template) of spatiotemporal data (5-10 sec duration) and calculates sliding-window correlation (SWC) between the template and the entire time-course, identifying time points of high correlation (with a set threshold arrived at with bootstrapping; which is 0.2 in the below figure). Supplementary Figure 2B illustrates the process of identifying the appearance of the QPP from a representative rsfMRI time-series dataset. The spatiotemporal blocks centered on these time points are then averaged to create a revised template for that QPP, and this process is repeated until the template converges

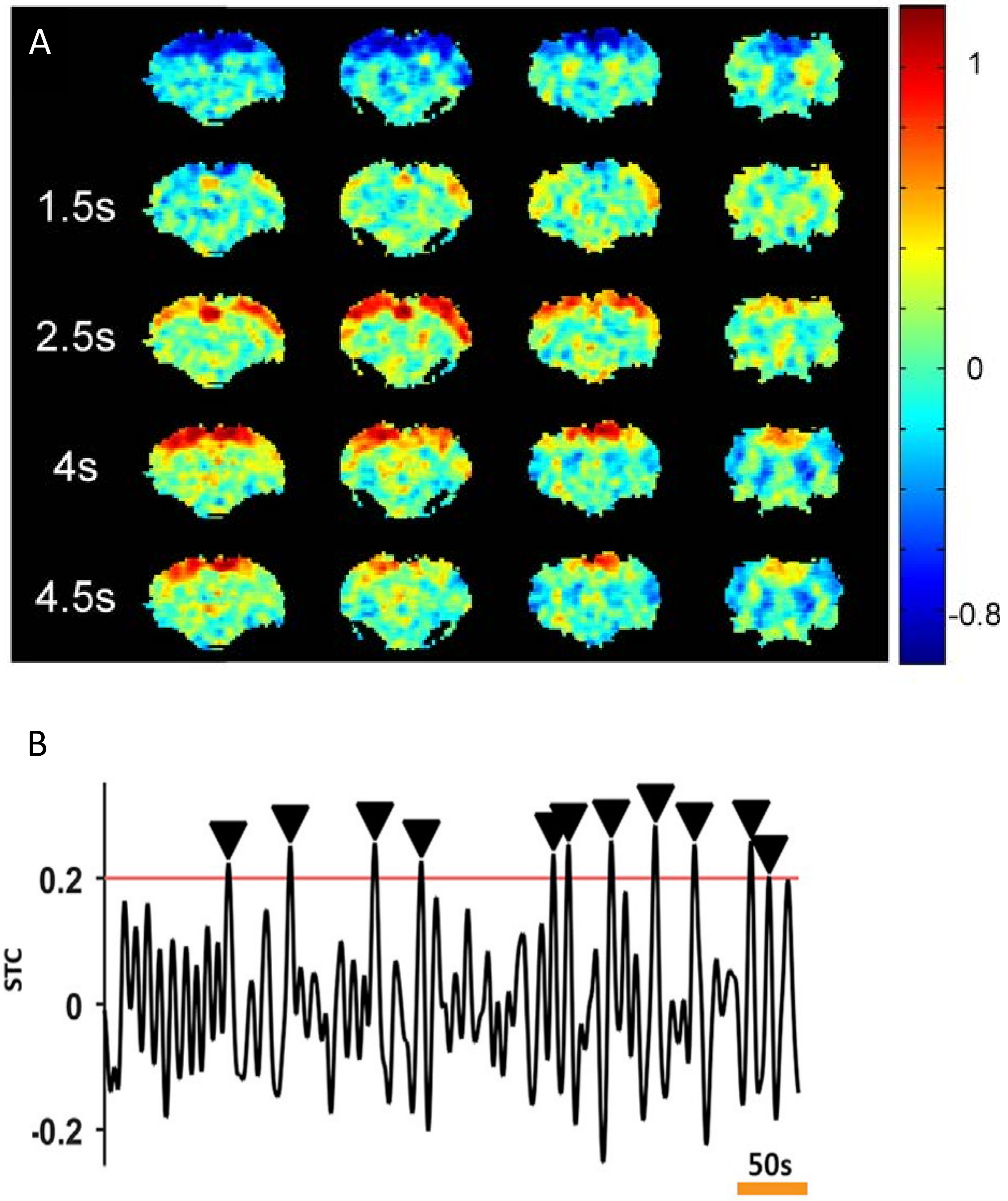

Supplementary Figure 2: (A) Example quasiperiodic pattern (QPP) template. The propagation of QPP is shown at different times past onset. The color bar represents STC. (B) Sliding template correlation of a QPP template in a representative time-series, showing instances of appearance of the QPP

Supplementary Methods: IHFC analysis

IHFC was examined on seed ROIs obtained from Paxinos atlas. The ROIs are listed below

Supplementary Table 1: Paxinos Regions of Interest (ROIs)

Paxinos ROI	Region of interest	Paxinos ROI	Region of interest
AID right	dorsal agranular insular area	PtPD right	Parietal cortex, posterior area, dorsal part
AIP right	posterior agranular insular area	PtPR right	Parietal cortex, posterior area, rostral part
AIV right	ventral agranular insular area	RSD right	dysgranular cortex
APir right	amygdalopiriform transition area	RSGb right	Retrosplenial granular cortex, B region
Au1 right	primary auditory area	RSGc right	Retrosplenial granular cortex, C region
AUD right	dorsal secondary auditory cortex	S1 right	primary somatosensory cortex
AuV right	ventral secondary auditory cortex	S1BF right	somatosensory 1, barrel field
Cg1 right	cingulate cortex, area 1	S1DZ right	somatosensory 1, dysgranular region
Cg2 right	cingulate cortex, area 2	S1DZ0 right	somatosensory 1, dysgranular zone 0
DI right	dysgranular insular cortex	S1FL right	somatosensory 1, forelimb region
DIEnt right	dorsintermed entorhinal cortex	S1HL right	somatosensory 1, hindlimb region
DLEnt right	dorsolateral entorhinal cortex	S1J right	somatosensory 1, jaw region
DLO right	dorsolateral orbital cortex	S1Sh right	somatosensory 1, shoulder region
Ect right	ectorhinal cortex	S1Tr right	somatosensory 1, trunk region
Fr3 right	Frontal cortex, area 3	S1ULp right	somatosensory 1, upper lip region
GI right	granular insular cortex	S2 right	secondary somatosensory cortex
GIDI right	granular and dysgranular insular cortex;	TeA right	temporal cortex, association area
LPtA right	lateral parietal association cortex	V1 right	primary visual cortex
M1 right	primary motor cortex	V1B right	primary visual cortex, binocular
M2 right	supplementary motor cortex	V1M right	primary visual cortex, monocular
MEnt right	entorhinal cortex, medial part	V2L right	visual cortex 2, lateral part
MPtA right	medial parietal association cortex	V2ML right	visual cortex 2, mediolateral part
PRh right	perirhinal cortex	V2MM right	visual cortex 2, mediomedial part
PtPC right	Parietal cortex, posterior area, caudal part	VIEnt right	Ventrintermed entorhinal cortex

Supplementary Results: TTA-P2 induced effects on QPPs persisted when estimated without GSR:

Supplementary Figure 3 (top) shows a QPP with anterior to posterior propagation estimated without performing global signal regression. The fluctuations in the strength of the QPP before and after injection of TTA-P2 is shown on the bottom. TTA-P2 administration significantly (paired t-test $p < 0.05$) reduced the strength (mean of positive STC values) of QPPs compared to Baseline. All the rats exhibited suppression of QPPs after TTA-P2 administration. The amount of suppression of QPPs induced by TTA-P2 varied from yielded 8-37% (mean 27%) across the rats.

Supplementary Figure 3: (top) Eight frames from the QPP template obtained from both groups' pre-injection concatenated functional timeseries (window length = 10s). (bottom) The QPP strength changes for each rat in TTA-P2 group. The values are estimated as the mean of positive excursions of the STC curve above zero, normalized by the maximum correlations for each subject.

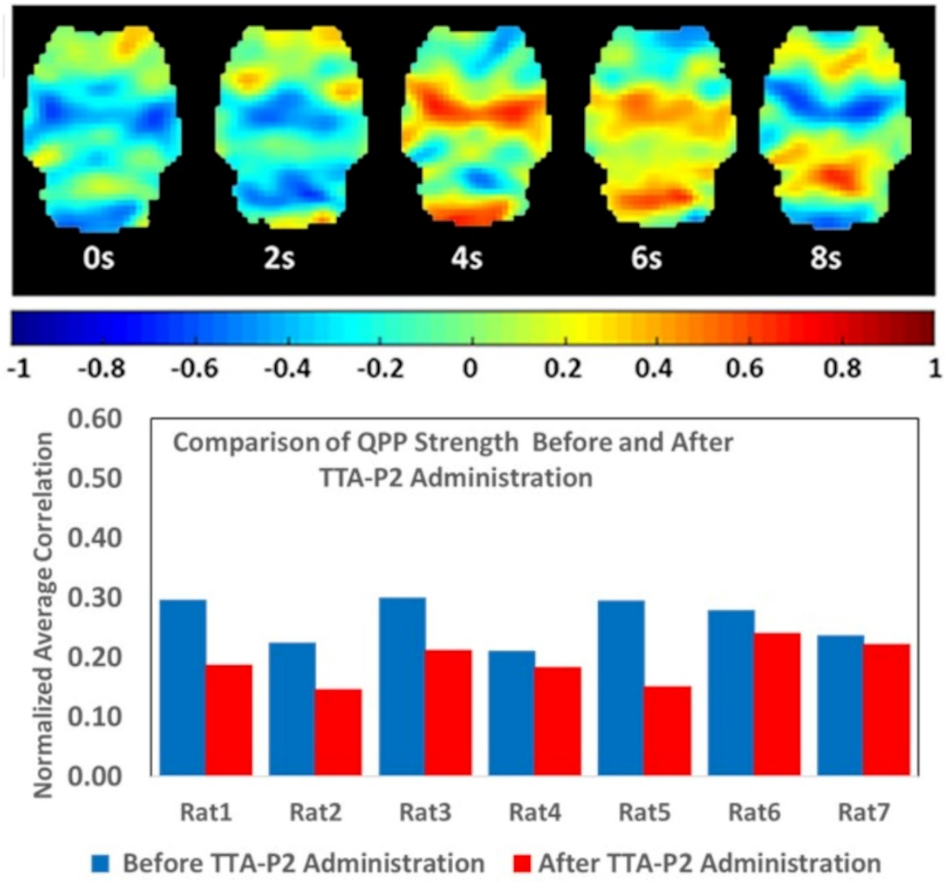

Supplementary Results: TTA-P2 administration induced increases IHFC are Reproducible:

As mentioned in the manuscript thirty-one of the remaining forty ROIs exhibited significantly (paired t-test $p < 0.05$) increased IHFC after injection of TTA-P2. Reproducibility of the IHFC increases varied between 86% to 100% (mean 93%) among the IHFCs of the ROIs which exhibited significant increase in IHFC after TTA-P2 administration (Supplementary Figure 4).

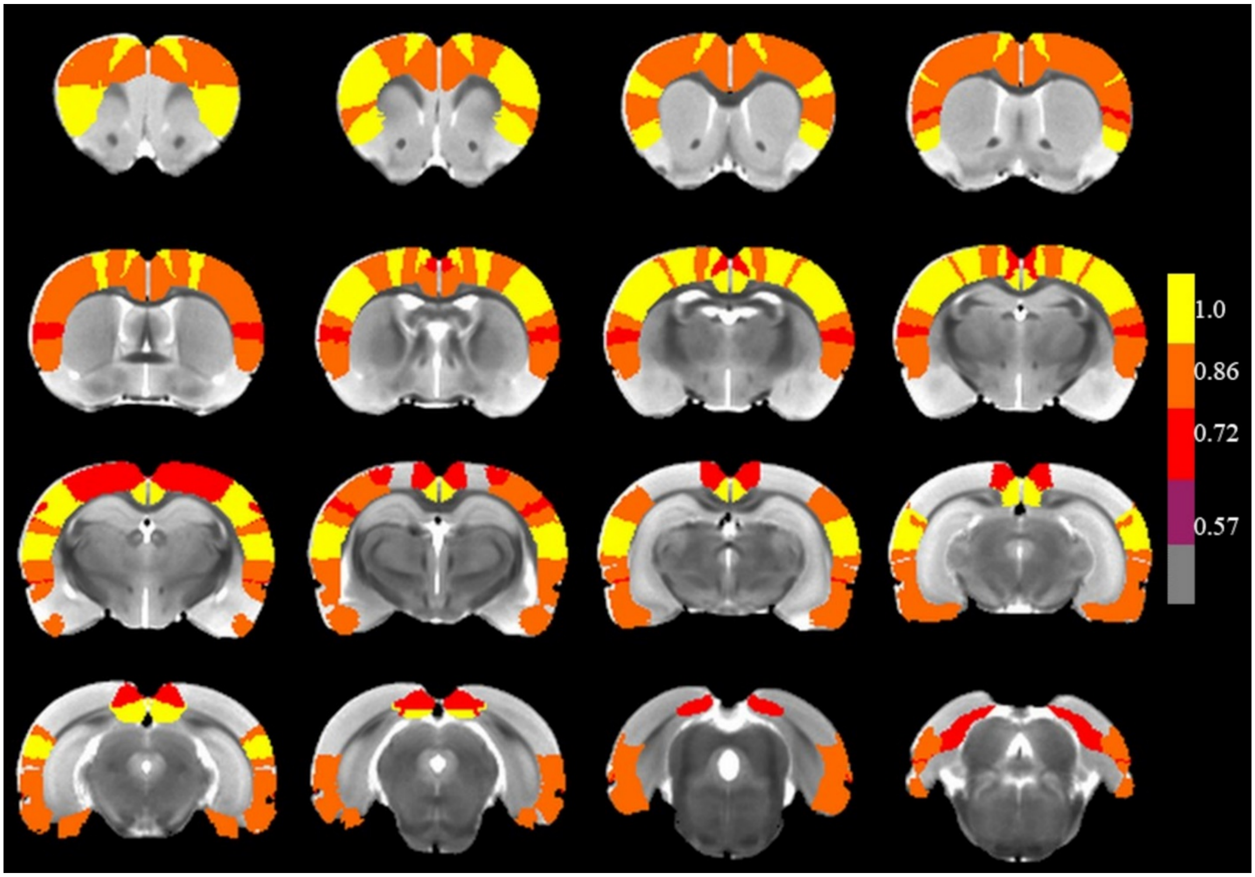

Supplementary Figure 4: Reproducibility of changes in IHFC after TTA-P2 administration in all 40 Paxinos ROIs overlaid on Paxinos Atlas brain.

TTA-P2 administration also increased IHFC calculated with GSR

Analyzing IHFC in Paxinos ROIs GSR yielded TTA-P2 engendered enhancements in IHFC in 22 ROIs after FDR correction (Supplementary Table 2).

Supplementary Table 2: TTA-P2 vs Baseline IHFC paired t-test after global signal regression for different Paxinos ROIs.

Paxinos ROI	t	p	FDR α	Paxinos ROI	t	p	FDR α
AID right	4.209	0.006	0.02	PtPD right	4.038	0.007	0.021
AIP right	2.57	0.042	0.056	PtPR right	4.443	0.004	0.02
AIV right	6.048	0.001	0.017	RSD right	2.011	0.091	0.104
APir right	NA	NA	NA	RSGb right	1.079	0.322	0.322
Au1 right	3.29	0.017	0.036	RSGc right	1.597	0.161	0.17
AUD right	4.266	0.005	0.02	S1 right	5.687	0.001	0.017
AuV right	2.126	0.078	0.091	S1BF right	3.277	0.017	0.036
Cg1 right	3.056	0.022	0.039	S1DZ right	3.002	0.024	0.039
Cg2 right	3.192	0.019	0.038	S1DZ0 right	1.805	0.121	0.131
DI right	3.258	0.017	0.036	S1FL right	3.944	0.008	0.022
DIEnt right	NA	NA	NA	S1HL right	4.796	0.003	0.02
DLEnt right	2.544	0.044	0.057	S1J right	1.918	0.104	0.115
DLO right	2.623	0.039	0.054	S1Sh right	NA	NA	NA
Ect right	3.821	0.009	0.023	S1Tr right	5.168	0.002	0.02
Fr3 right	2.289	0.062	0.078	S1ULp right	3.047	0.023	0.039
GI right	2.891	0.028	0.041	S2 right	3.562	0.012	0.03
GIDI right	NA	NA	NA	TeA right	2.633	0.039	0.054
LPtA right	4.809	0.003	0.02	V1 right	6.126	0.001	0.017
M1 right	2.956	0.025	0.039	V1B right	3.124	0.02	0.039
M2 right	4.427	0.004	0.02	V1M right	4.026	0.007	0.021
MEnt right	NA	NA	NA	V2L right	4.277	0.005	0.02
MPtA right	2.21	0.069	0.084	V2ML right	2.967	0.025	0.039
PRh right	NA	NA	NA	V2MM right	1.458	0.195	0.2
PtPC right	NA	NA	NA	VIEnt right	NA	NA	NA

dof = degrees of freedom; FDR p = false discovery rate corrected p

Supplementary Results: Functional Connectivity of rat barrel and auditory cortices at Baseline

The FC of right S1BF and right auditory cortex was examined at Baseline before administration of the drug. S1BF exhibited (Supplementary Figure 5) significant (1-sample t-test FWE $\alpha < 0.05$) in a lot of areas which exhibited increases in FC after TTA-P2 administration: including bilateral somatosensory, motor, auditory, visual, and parietal cortices. Auditory cortex also exhibited significant FC with regions found to exhibit increased FC after TTA-P2 administration (Supplementary Figure 6)

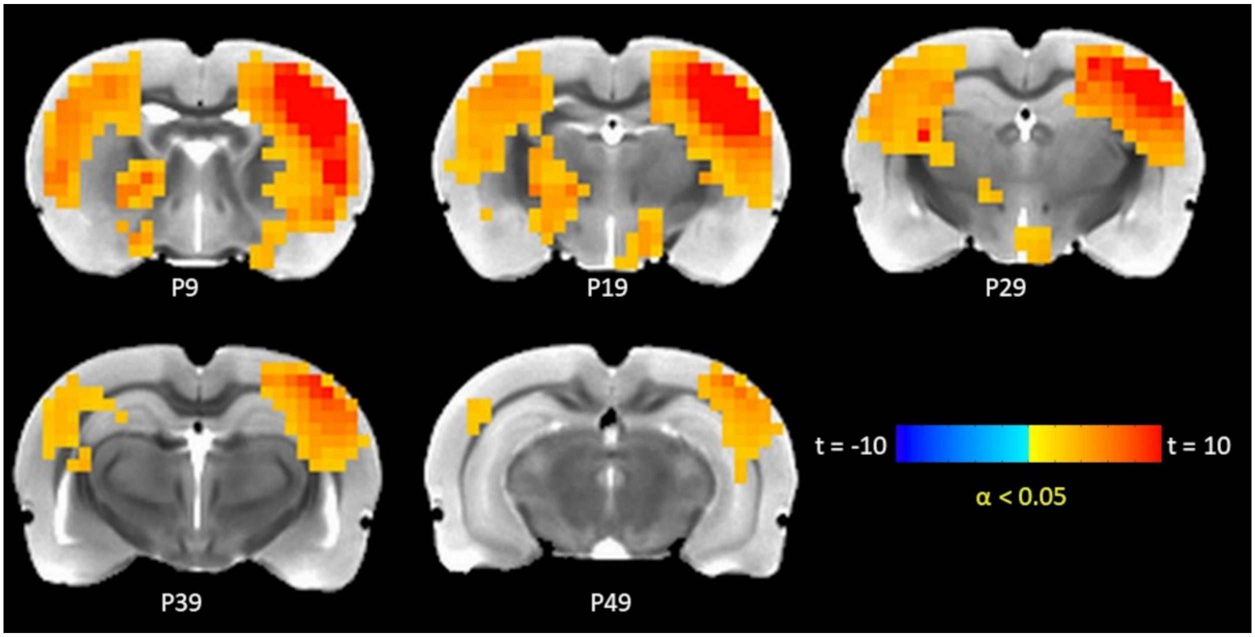

Supplementary Figure 5: Baseline condition 1-sample t-statistic map highlighting regions with significant (1-sample t-test FWE $\alpha < 0.05$) FC to right S1-BF ROI. The slice-location co-ordinates are in Paxinos space. Left hemisphere is on the left-hand side of the maps

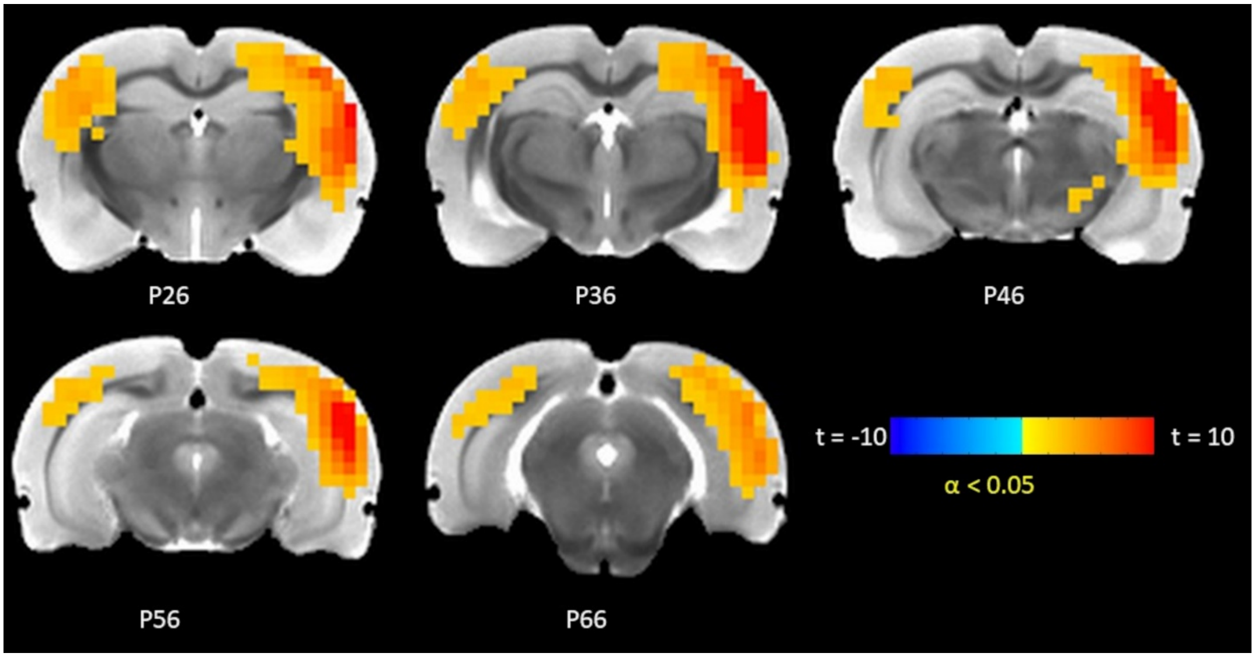

Supplementary Figure 6: Baseline condition 1-sample t-statistic map highlighting regions with significant (1-sample t-test FWE $\alpha < 0.05$) FC to right auditory cortex ROI. The slice-location co-ordinates are in Paxinos space. Left hemisphere is on the left-hand side of the maps

TTA-P2 administration increased FC (estimated with GSR) to rat barrel and auditory cortices

When global signal regression was employed during preprocessing TTA-P2 significantly (paired t-test FWE $\alpha < 0.05$) increased the rsfMRI FC between both right S1-BF (Supplementary Figure 7) and right auditory cortex (Supplementary Figure 8) in similar but not all areas which showed enhanced FC in TTA-P2 condition when estimated without GSR.

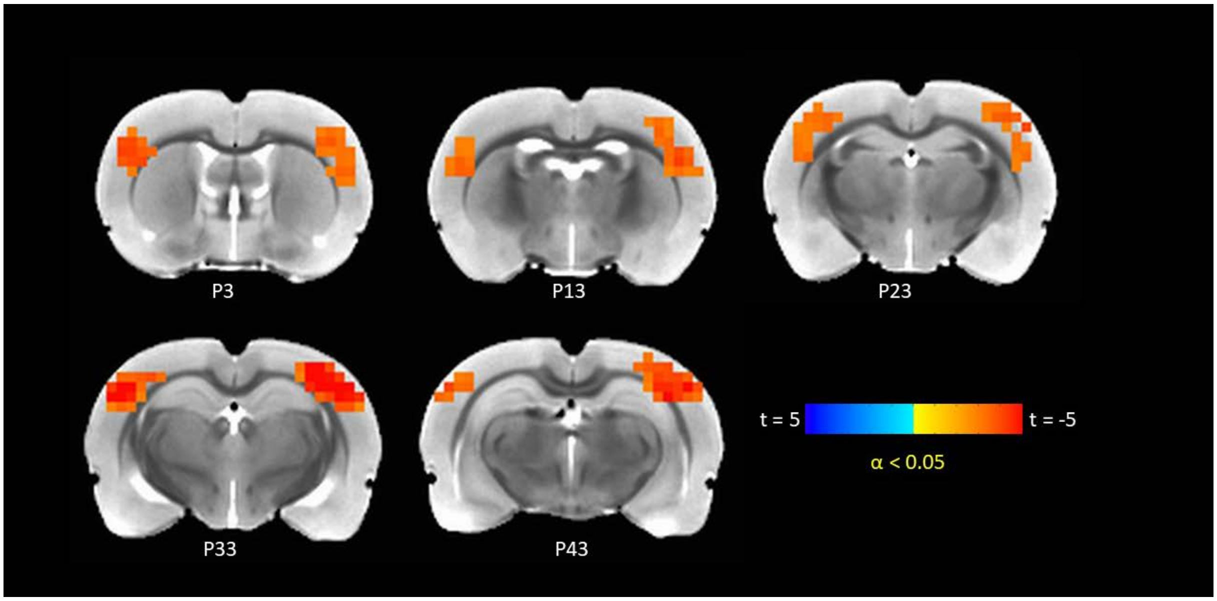

Supplementary Figure 7: TTA-P2 vs Baseline t-statistic maps highlighting regions with enhanced FC (estimated while employing global signal regression) to right S1-BF ROI after TTA-P2 administration. The slice-location co-ordinates are in Paxinos space. Left hemisphere is on the left-hand side of the maps

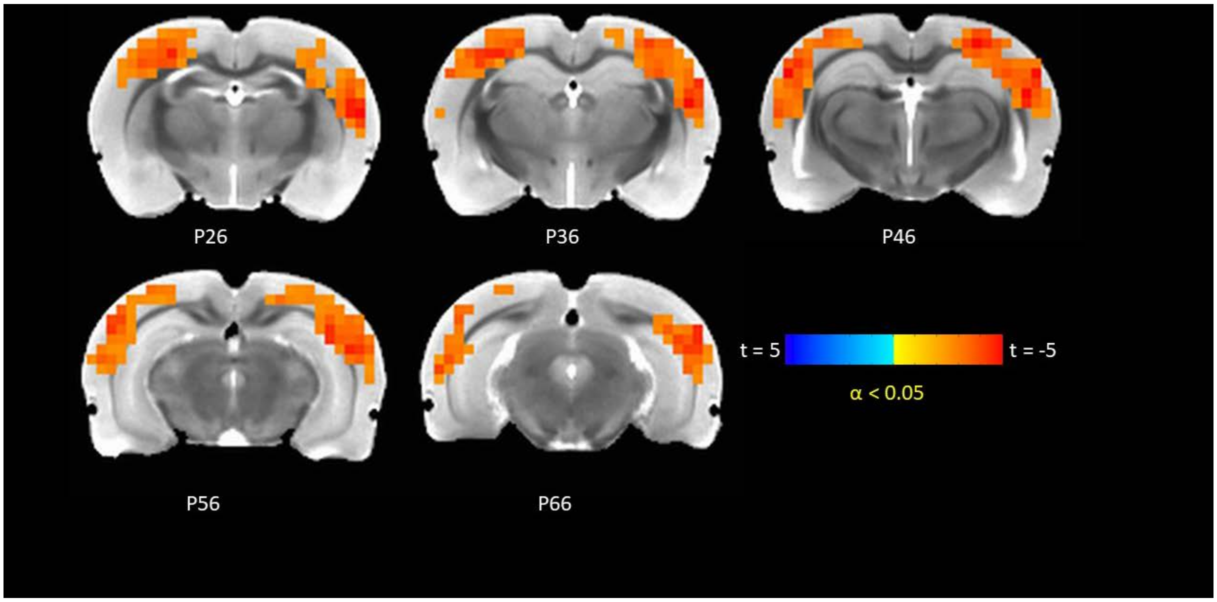

Supplementary Figure 8: TTA-P2 vs Baseline t-statistic maps highlighting regions with enhanced FC (estimated while employing global signal regression) to right auditory cortex ROI after TTA-P2 administration. The slice-location co-ordinates are in Paxinos space. Left hemisphere is on the left-hand side of the map
